# TrkC-dependent regulation of dendritic branching shapes dendritic bundle architecture in the rat granular retrosplenial cortex

**DOI:** 10.3389/fncir.2026.1925955

**Published:** 2026-09-24

**Authors:** Toshio Miyashita

**Affiliations:** Department of Anatomy, Teikyo University School of Medicine, Tokyo, Japan

**Keywords:** cortical module, dendrite, layer 1, minicolumn, NT-3

## Abstract

Cortical modules are thought to provide anatomical frameworks for local microcircuits. However, the mechanisms regulating the morphological organization of these modules during development remain poorly understood. In the rat granular retrosplenial cortex, layer 1 contains prominent dendritic bundle-centered modules formed by the apical dendrites of layer 2 neurons and aligned with specific afferent inputs. I investigated the molecular mechanisms regulating the architecture of these dendritic bundles, focusing on TrkC, a high-affinity receptor for neurotrophin-3. Reanalysis of developmental gene expression data showed that neurotrophin-3 was transiently enriched in layer 2 before dendritic bundle formation, whereas TrkC expression was maintained at above-average levels and became modestly elevated after bundle formation. Pathway analysis further suggested developmental changes in neurotrophin pathways. To examine whether perturbation of TrkC-related signaling affects dendritic bundle architecture, I overexpressed a truncated TrkC construct lacking the intracellular tyrosine kinase domain in layer 2 neurons via in utero electroporation. Overexpression of this construct expanded dendritic bundle territories in upper layer 1. Sparse single-cell labeling revealed that this bundle-level phenotype was accompanied by increased branching of the apical dendrites of individual layer 2 neurons. These findings suggest that TrkC-related signaling may regulate the morphology of dendritic bundles by restricting excessive branching of apical dendrites during development. Thus, regulation of dendritic branching may contribute to the refinement of the distinctive dendritic bundle architecture of the granular retrosplenial cortex.

## Introduction

Cortical modules have been proposed to serve as functional units of the cerebral cortex. The cortex is organized into layers along the radial axis, as well as into columnar and modular architectures that integrate neurons, dendritic compartments, and layer-specific afferent inputs (Rockland and Ichinohe, 2004; Horton and Adams, 2005; Rockland, 2010; Miyashita, 2025; Defelipe et al., 2012). These local architectures are believed to contribute to the formation of functional microcircuits; however, their anatomical organization and developmental mechanisms remain incompletely understood.

The granular retrosplenial cortex (gRSC), which is involved in spatial memory, learning, and navigation, is particularly well developed in rodents and exhibits a distinctive yet enigmatic structure (Stacho and Manahan-Vaughan, 2022; Alexander et al., 2023). This cortical region contains a conspicuous modular architecture in layer (L)1. In rats, the apical dendrites of the callosally projecting L2 pyramidal neurons form dendritic bundles in L1 (Wyss et al., 1990). These bundles are interdigitated with OCAM-positive modules composed of the apical dendritic tufts of L5 pyramidal neurons (Ichinohe et al., 2003). Moreover, these postsynaptic domains are associated with distinct presynaptic inputs: L2 dendritic bundle domains are associated with vGluT2-positive thalamocortical terminals, whereas OCAM-positive domains overlap with vGluT1-positive corticocortical terminals. Thus, L1 of the gRSC contains a higher-order modular architecture composed of aligned postsynaptic dendritic compartments and presynaptic input domains (Ichinohe, 2012; Miró-Bernié et al., 2005). Within this architecture, dendritic bundles derived from L2 neurons may serve as critical hubs for local microcircuit organizations.

Despite these prominent anatomical features, the developmental mechanisms underlying dendritic bundle formation in the gRSC remain unclear. Viale et al. (2019) demonstrated that the early postnatal knockdown of neurotrophin-3 (NT-3) in a subset of gRSC L2 neurons causes persistent dendritic defects and leads to long-lasting abnormalities in spatial exploration, navigation, and memory. These findings suggest that the modular architecture of the gRSC provides an important anatomical substrate for adult spatial behavior. However, little is known about the molecular signals that regulate dendritic development and the refinement of bundle morphology in these neurons.

In our previous study, gene expression analysis of laser-microdissected cortical microtissues revealed that *Ntf3* is transiently and specifically expressed in the gRSC L2 before dendritic bundle formation (Miyashita et al., 2010). Neurotrophins, including NT-3, regulate dendritic growth and branching in various neuronal populations. Their effects depend strongly on the cortical area, layer, cell type, and developmental stage. For example, in the ferret visual cortex, different neurotrophins exert layer-specific and sometimes opposing effects on dendritic growth (McAllister et al., 1995; McAllister et al., 1997; McAllister et al., 1999). Based on these findings, I hypothesized that NT-3 contributes to dendritic bundle formation in gRSC. Consistent with this idea, the ectopic expression of NT-3 in L2 of the primary somatosensory (S1) barrel cortex, which normally lacks such dendritic bundles, induces dendritic bundle-like structures resembling those observed in the gRSC (Miyashita et al., 2010).

However, whether secreted NT-3 acts on the NT-3-expressing L2 neurons themselves and thereby influences the developmental organization of the dendritic bundles formed by these neurons remains unclear. TrkC is a high-affinity receptor for NT-3 that has been implicated in dendritic growth and branching in several neuronal populations, including cerebellar Purkinje cells (McAllister et al., 1995; McAllister et al., 1997; Joo et al., 2014). Therefore, I focused on TrkC and examined whether perturbation of TrkC-related signaling affects the architecture of L2 dendritic bundles in the gRSC. To examine this possibility, I overexpressed a TrkC variant lacking the tyrosine kinase domain (TrkC-dTK) in gRSC L2 neurons using in utero electroporation. I found that overexpression of TrkC-dTK increased dendritic branching and expanded dendritic bundle territories, suggesting that TrkC-related signaling may contribute to the refinement of dendritic bundle architecture by restricting excessive dendritic branching.

## Methods

### Microarray data analysis

Gene expression data from eight GeneChip Rat Genome 230 2.0 arrays (Miyashita et al., 2010) were used in this study. These arrays represent four experimental conditions, each with two biological replicates: gRSC L2, gRSC L5, and L2 of the somatosensory cortex barrel field (SBF) at postnatal day (P) 3, as well as gRSC L2 at P12. The samples were obtained from laser-microdissected rat cerebral cortical tissues that differed in postnatal age, cortical area, and cortical layer.

CEL files were analyzed using R/Bioconductor and RMA-normalized expression values were used for further analyses. For the analysis of *trkC* expression, two *trkC* probe sets corresponding to the 5′ region (1377308_a_at) and the 3′ region (1368939_a_at) of the transcript were examined separately. The expression levels of *Ntf3* were also analyzed for comparison. The mean and standard deviation of the RMA-normalized expression values across the GeneChip dataset were calculated to evaluate whether TrkC expression was above the general expression level in the dataset. For z-score visualization of *trkC* and *Ntf3* expression, the expression values were normalized across the eight arrays.

For pathway-level analysis, genes included in the KEGG Neurotrophin signaling pathway (rno04722) were extracted and analyzed. Biological duplicate samples were averaged for the four selected conditions, and gene-wise z-score-normalized expression profiles were visualized as a heatmap with hierarchical clustering.

Principal component analysis (PCA) was performed using the expression profiles of rno04722 to evaluate sample separation under the four conditions. Genes contributing to PC1 and PC4 were ranked based on the PCA loading. Highly loaded genes were subjected to GO and KEGG pathway enrichment analyses.

Condition-level similarity was further assessed using pairwise correlation distances calculated as 1 − Pearson’s correlation coefficients from the z-score-normalized expression profiles of the rno04722 genes. The resulting distance matrix was visualized using multidimensional scaling and an unrooted neighbor-joining tree.

### Plasmid construction

The pCAG-TrkCdTK-GFP plasmid used for *in utero* electroporation was constructed as follows. Reverse transcription Polymerase chain reaction (RT-PCR) was performed using the following oligonucleotide primers:

*trkC*met; 5’-GAAGCAGAATTCGGAG**ATG**GATGTC-3′, and *trkC*JM, 5-TTGCTCGAGTGTGTCTGGCTTGTGGCAA-3′ (underlined sequences indicate the XhoI site used for further construction, and bold indicates the translation initiation site). The amplified DNA fragment was then subcloned into the pGEM-T Easy Vector (Promega, Madison, WI, USA) to generate pGEM-TrkCdTK, which contains the *trkC* gene before its kinase domain and translates to the TrkC protein with 528 amino acid residues. GFP DNA fragments were amplified from the pCAGGS-GFP plasmid DNA as a template using the following set of primers:

*gfp*5xho; 5’-CGGTCCTCGAG**ATG**GTGAGCAAG-3′, and *gfp*3Eco, 5’-GTCGCGGAATTCTTACTTGTACAGC-3′ (underlined sequences indicate the XhoI and EcoRI sites used for further construction, and bold indicates the codon for the 1st Met of GFP). The amplified DNA fragment was then subcloned into the pGEM-T Easy Vector to generate pGEM-GFP. SpeI/XhoI fragments from pGEM-TrkCdTK and XhoI/EcoRI fragments from pGEM-GFP were subcloned into the SpeI/EcoRI sites of the pBluescript II plasmid to create pBS-TrkCdTK. The SpeI/EcoRI DNA fragments excised from pBS-TrkCdTK were blunt-ended and subcloned into the blunted XhoI site of the pCAGGS plasmid (Niwa et al., 1991) to create pCAG-TrkCdTK-GFP.

### In utero electroporation

Pregnant Wistar rats (18 days post coitum) were anesthetized using isoflurane and subjected to an abdominal incision to expose the uterus. Embryos were visualized through the uterine wall, and plasmid solutions mixed with 0.05% fast green dye were injected into the lateral ventricle through a glass capillary. Electric pulses were delivered to the embryos by gently clasping their heads with forcep-shaped electrodes connected to a square-pulse electroporator. Five 50 V pulses of 50 ms were applied at 950 ms intervals. The uterine horns were repositioned in the abdominal cavity, and the abdominal wall and skin were sutured. The animals recovered and their pups were born by natural delivery. The pups were reared until P25 and perfused as described below. Plasmids were used at concentrations of 1–1.5 μg/μL unless otherwise indicated. To express the TrkC-dTK-GFP fusion protein, pCAG-TrkCdTK-GFP was used, whereas pCAGGS-GFP was used in the control experiment. Sparse-labeling experiments were performed using pCAG-LoxP-STOP-LoxP-mCherry together with a titrated amount of pCAG-Cre (0.1 ng/μL), enabling visualization of individual neuronal morphologies (Hatanaka and Yamauchi, 2013; Matsui et al., 2013).

### Perfusion fixation and brain tissue sectioning

Rats were anesthetized and perfused transcardially with 0.9% saline for 1 min followed by 4% paraformaldehyde (PFA) in 0.1 M phosphate buffer (PB, pH 7.4) for 10 min. The dissected brains were postfixed in the same fixative for 2 h. The brains were then immersed in 30% sucrose for cryoprotection for 20–40 h at 4 °C and cut on a freezing microtome into coronal sections (40 μm thick) or sagittal sections (30 μm thick), the latter yielding tangential sections of the gRSC. gRSC sections located 2.7–3.5 mm caudal to Bregma were used for analysis.

### Riboprobe preparation and *in situ* hybridization

The sense and antisense RNA probes for *trkC* were synthesized using a digoxigenin (DIG)-RNA labeling kit with SP6 or T7 RNA polymerase (Merck). Cortical tissue sections, including gRSC sections, from Wistar rat pups (P3, P10, and P14) were used for *in situ* hybridization. The sections were mounted on MAS-coated slides (Matsunami, Osaka, Japan). Standard methods were used for *in situ* hybridization of slide-mounted histological sections, as previously described (Miyashita et al., 2010). No signals were observed when sections were processed using DIG-labeled sense RNA probes.

### Immunofluorescent staining

Free-floating sections were incubated for 1 h in 0.1 M phosphate-buffered saline (PBS) containing 0.5% Triton X-100 and 5% normal donkey serum (PBS-TD) at room temperature. The sections were then incubated for 40–48 h at 4 °C with a combination of two of the following diluted primary antibodies (goat anti-OCAM (R&D systems, Cat# AF778) 1:300; mouse anti-mCherry(16D7) (Thermo Fisher Scientific, Cat# M11217) 1:2000; chicken anti-GFP (Abcam, Cat# ab13970) 1:500). After rinsing with PBS, the sections were placed in PBS-TG containing fluorescently labeled secondary antibodies (Cy3–conjugated anti-mouse IgG, Cy3–conjugated anti-goat IgG, or Alexa 488–conjugated anti-chicken IgG; 1:500).

Fluorescently labeled tissue sections were scanned using a NanoZoomer XR (Hamamatsu) or a confocal microscope (A1, Nikon).

### Data analysis

#### Tangential cross-sectional area of dendritic bundles

Tangential sections (30 μm thick) from 14 animals were used for the analysis: four control animals expressing GFP and ten animals expressing dominant-negative TrkC and TrkC-dTK-GFP. Double immunofluorescence signals for GFP and OCAM were acquired using a NanoZoomer XR scanner (Hamamatsu). GFP was visualized using Alexa Fluor 488, and OCAM immunoreactivity was visualized using Cy3.

I analyzed 35 dendritic bundles from control gRSC samples and 135 dendritic bundles from TrkC-dTK-GFP-expressing gRSC samples. In coronal sections, I confirmed that GFP-positive dendritic bundles were located within the OCAM-negative domains in L1. In utero electroporation labeled only approximately 40–60% of L2 neurons; therefore, the area occupied by GFP-labeled dendrites did not necessarily represent the full extent of each dendritic bundle. Accordingly, OCAM-negative domains containing GFP-positive dendritic bundles were used as anatomical landmarks for putative dendritic bundle territories.

Serial tangential sections were manually aligned using the blood vessels as reference points. Sections containing GFP-positive L2 cell bodies were defined as section 1 (S1), and progressively more superficial sections were designated as section 2 (S2) and onward. OCAM-negative domains containing GFP-positive dendritic bundles were manually outlined, and their areas were measured in pixels using the FIJI/ImageJ software. The area ratios between sections 3 and 4 and between sections 3 and 5 were calculated and compared between the control and TrkC-dTK-GFP-expressing samples.

#### Dendritic branch point analysis

L2 neurons in the gRSC, expressing only GFP or TrkC-dTK-GFP were sparsely labeled with mCherry and analyzed for dendritic branch points in 40-μm-thick tissue sections. Seventeen control neurons and 52 TrkC-dTK-GFP-expressing neurons were analyzed from four animals in each group for a total of eight rats.

Confocal z-stack images of mCherry-positive L2 neurons were used for single-cell analysis. Only neurons whose dendrites could be traced continuously and did not overlap with dendrites from neighboring labeled cells were included. The L1 portion of each apical dendrite was divided into five bins along the depth axis from the deepest region near L2 to the most superficial region near the pial surface. The number of dendritic branch points within each bin was counted and compared between the control GFP-expressing and TrkC-dTK-GFP-expressing neurons.

### Statistical analysis

The data for dendritic bundle area at each section and the area ratios between sections were logarithmically transformed because some groups deviated from normality. Normality of the log-transformed data was assessed using the Lilliefors test, and no significant deviation from normality was detected in any group (all *p* > 0.05).

The log-transformed data were compared between the control and TrkC-dTK groups using a two-sided Welch’s t-test. Holm correction was applied separately to the three comparisons of bundle area (S3, S4, and S5) and the two comparisons of area ratios (S4/S3 and S5/S3).

Branching point counts were not normally distributed and were therefore compared between the control and TrkC-dTK groups using a two-sided Mann–Whitney U test. For the bin-wise analysis, the control and TrkC-dTK groups were compared within each bin, with Bonferroni correction for the five comparisons.

Data from sections physically lost during tissue processing were treated as missing values and excluded from analyses requiring measurements from those sections. Missing values were not imputed. The specific statistical tests and significance levels are indicated in the corresponding figures and figure legends.

## Results

### TrkC expression in the granular retrosplenial cortex during postnatal development

In our previous study, we reported that NT-3 is specifically and strongly expressed in L2 of the gRSC during dendritic bundle formation (Miyashita et al., 2010). Therefore, I investigated how the expression of TrkC, a receptor for NT-3, changes during postnatal development in relation to dendritic bundle formation. To this end, I reanalyzed the GeneChip data from our previous study.

I compared expression levels across four conditions: gRSC L2, gRSC L5, and SBF L2 at P3, the stage before dendritic bundle formation, as well as gRSC L2 at P12, the stage after dendritic bundle formation. Each condition was represented by two biological replicates, yielding a total of eight samples.

Consistent with our previous report, *Ntf3* showed temporal-, regional-, and laminar-specific expression, with strong expression in gRSC L2 at P3. In contrast, *trkC* was expressed at relatively stable levels across the four conditions, with expression values remaining above the mean level of the GeneChip dataset analyzed in this study. The RMA-normalized expression values of the *trkC* probe sets ranged from 7.11 to 10.53, whereas the mean expression value across the dataset was 6.27, with a standard deviation of 2.46. Among the two TrkC probe sets, the probe set corresponding to the 5′ region showed a modest increase in gRSC L2 at P12 (Figure 1A).

**Figure 1:**
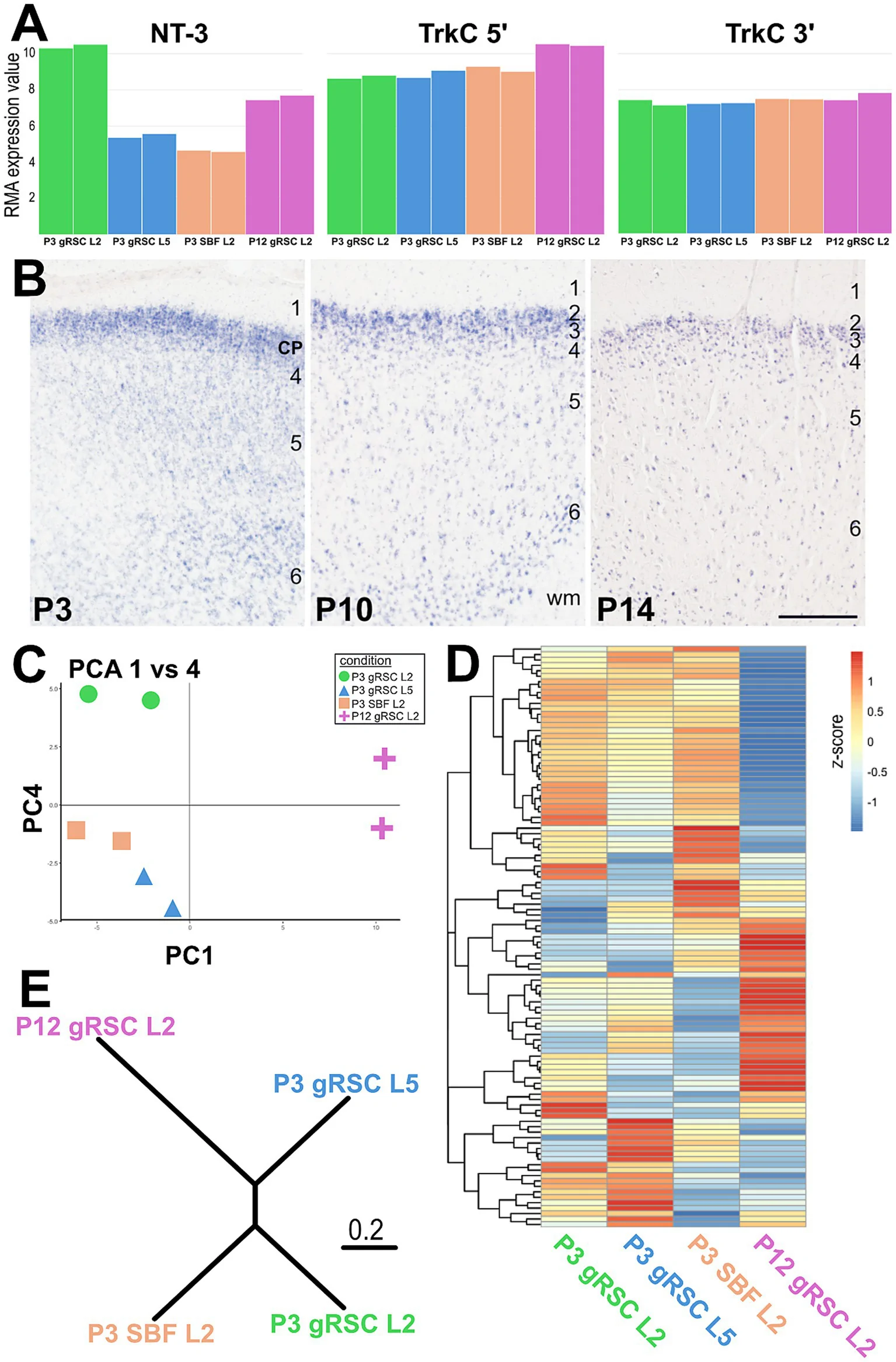
TrkC and NT-3 expression in the developing rat granular retrosplenial cortex (gRSC). Gene expression levels were analyzed using eight GeneChip Rat 230 arrays comprising four conditions with biological duplicates. **(A)** RMA-normalized expression values for the NT-3 probe set and two TrkC probe sets corresponding to the 5′ and 3′ regions are shown as individual bars for two biological replicates per condition across different ages, cortical layers, and cortical areas of the developing cerebral cortex. **(B)** In situ hybridization of TrkC in the gRSC at three different developmental stages. Scale bar, 100 μm. **(C)** Principal component analysis (PCA) of eight GeneChip datasets based on expression levels of genes in the KEGG neurotrophin signaling pathway (rno04722). The samples were plotted according to PC1 and PC4 scores. **(D)** Heat map showing the expression patterns of rno04722 genes under the four experimental conditions. The expression values were averaged across biological duplicates within each condition and converted to z-scores for each gene. Genes were ordered according to hierarchical clustering. **(E)** Unrooted tree showing the similarity of gene expression patterns in the neurotrophin signaling pathway among the four experimental conditions, constructed using correlation distances based on rno04722 gene expression profiles. The scale bar represents a correlation distance of 0.2.

I also examined expression patterns as z-scores calculated across the eight GeneChip arrays. Within the samples examined, this analysis showed a temporally complementary pattern between *Ntf3* and *trkC*. *Ntf3* expression was the highest in gRSC L2 at P3, whereas *trkC* expression was relatively higher in gRSC L2 at P12 (Supplementary Figure 1A).

*In situ* hybridization further confirmed that *trkC* was continuously expressed in the L2 of the gRSC at P3, P10, and P14, although the signal appeared to decrease slightly in L5 at P14 (Figure 1B). Conventional *in situ* hybridization is not fully quantitative thus, these data support the presence of above-average *trkC* expression in gRSC L2 during the formation of dendritic bundles. Additional GeneChip data from P12 and P28 gRSC also indicated higher *trkC* expression in L2 than in L5 (Supplementary Figure 1B).

Together, these expression analyses suggest that dendritic bundle formation and the specification of gRSC L2 neurons may be regulated by transient NT-3 expression during the early postnatal period. This occurs in a region where its receptor, TrkC, is maintained at above-average expression levels and becomes modestly elevated after dendritic bundle formation.

### Neurotrophin signaling pathway genes characterize the developmental and regional specificity of gRSC L2

The temporally complementary expression patterns of *Ntf3* and *trkC* (Supplementary Figure 1A) suggest that neurotrophin-related intracellular signaling may be regulated in a developmental stage-, region-, and layer-specific manner during gRSC dendritic bundle formation. Therefore, I analyzed the expression dynamics of neurotrophin-related molecules using GeneChip data from four experimental conditions. When I investigated the expression profiles of genes associated with the neurotrophin signaling pathway (KEGG rno04722) under each condition, distinct condition-specific patterns were observed. I then performed PCA using this gene set (KEGG rno04722; Figure 1C). PC1 separated P12 gRSC L2 from the other P3 samples. *trkC* (*Ntrk3*) was among the genes that showed high positive loading for PC1. KEGG pathway enrichment analysis of the top 20 genes with positive PC1 loadings revealed that eight, including *trkC* were associated with the calcium signaling pathway (rno04020; Table 1). In contrast, PC4 distinguished P3 gRSC L2 from the other samples, with *Ntf3* among the genes showing high positive loadings for PC4 (Table 2).

**Table 1:** The top 20 genes with positive PC1 loadings.

Rank	Gene	Loading_PC1
1	*Camk2a	0.141706074
2	Map2k1	0.139717285
3	*Ntrk3	0.135776051
4	Pdpk1	0.132736034
5	Foxo3	0.130741812
6	*Camk4	0.127847328
7	*Ntrk2	0.124747647
8	Matk	0.123689042
9	Irak2	0.119809412
10	*Ngf	0.119719009
11	Bdnf	0.116875943
12	Mapk11	0.114032354
13	*Calm1	0.110349836
14	*Calml3	0.108516161
15	Arhgdig	0.104401972
16	Map3k5	0.101834066
17	Sort1	0.10036102
18	*Camk2d	0.099562269
19	Shc1	0.099001142
20	Ngfr	0.095660561

*Genes associate with Calcium signaling pathway (rno04020).

**Table 2:** The top 20 genes with positive PC4 loadings.

Rank	Gene	Loading_PC4
1	Ntf3	0.254662079
2	Ripk2	0.182915242
3	Braf	0.174634561
4	Bdnf	0.167578073
5	Rps6ka5	0.161214289
6	Gab1	0.156715233
7	Ngfr	0.146151036
8	Rap1b	0.143157305
9	Mapk11	0.12033466
10	Pik3ca	0.120067697
11	Sh2b3	0.12004802
12	Foxo3	0.118610783
13	Pik3r3	0.117672014
14	Map3k1	0.112329492
15	Map3k3	0.110788555
16	Tp53	0.110382746
17	Frs2	0.105440144
18	Zfp110	0.104034344
19	Akt2	0.103925249
20	Gsk3b	0.102421186

For genes included in the KEGG rno04722 neurotrophin signaling pathway, mean expression across biological duplicates was calculated and visualized as a heatmap after gene-wise z-score normalization. Hierarchical clustering analysis revealed that the rno04722 gene set appeared to segregate into four major gene clusters with distinct expression patterns, based on the similarity of expression profiles across the four conditions. The heatmap further showed clear condition-dependent expression patterns of neurotrophin signaling-related genes (Figure 1D).

The unrooted neighbor-joining tree showed the four conditions did not collapse into a single shared expression profile, but occupied distinct positions based on the expression patterns of neurotrophin signaling-related genes (Figure 1E). These findings suggest that the transcriptional state of the neurotrophin signaling pathway components may vary according to the cortical region, cortical layer, and developmental stage. In particular, the intracellular calcium signaling pathway may play a crucial role in establishing the specificity of gRSC L2 prior to and/or during dendritic bundle formation.

### Structure of cortical modules in L1 of the gRSC

In L1 of the gRSC, dendritic bundles are accompanied by surrounding structures, forming a modular architecture. Previous studies have shown that dendritic bundles labeled by MAP2, a pan-dendritic marker, are distributed in this region in a pattern complementary to that of OCAM, which is localized to the dendritic tufts of L5 pyramidal neurons in the gRSC (Ichinohe et al., 2003).

To examine the cellular organization of these modules in greater detail, I visualized L2 neurons and their dendrites using *in utero* electroporation (IUE) of a GFP-expressing plasmid. Electroporation was carried out at E18, (corresponding to the birthdate of neurons in the gRSC L2), and the detailed dendritic bundle structures were analyzed at P25.

In gRSC coronal sections, GFP-labeled L2 neurons extended their apical dendrites toward L1, where their dendrites formed prominent dendritic bundles. These dendritic bundles were mainly observed in L1b/c, whereas in the more superficial L1a, the GFP-labeled dendritic tufts extended laterally and occupied the sublayer more diffusely (Figure 2A). Thus, GFP labeling via IUE effectively visualized dendritic module structures, including the sublayer organization of L1 dendritic bundles in the gRSC. These results suggest that the previously described MAP2-labeled dendritic bundles correspond to the dendritic bundles formed by L2 pyramidal neurons that were labeled by IUE at E18.

**Figure 2:**
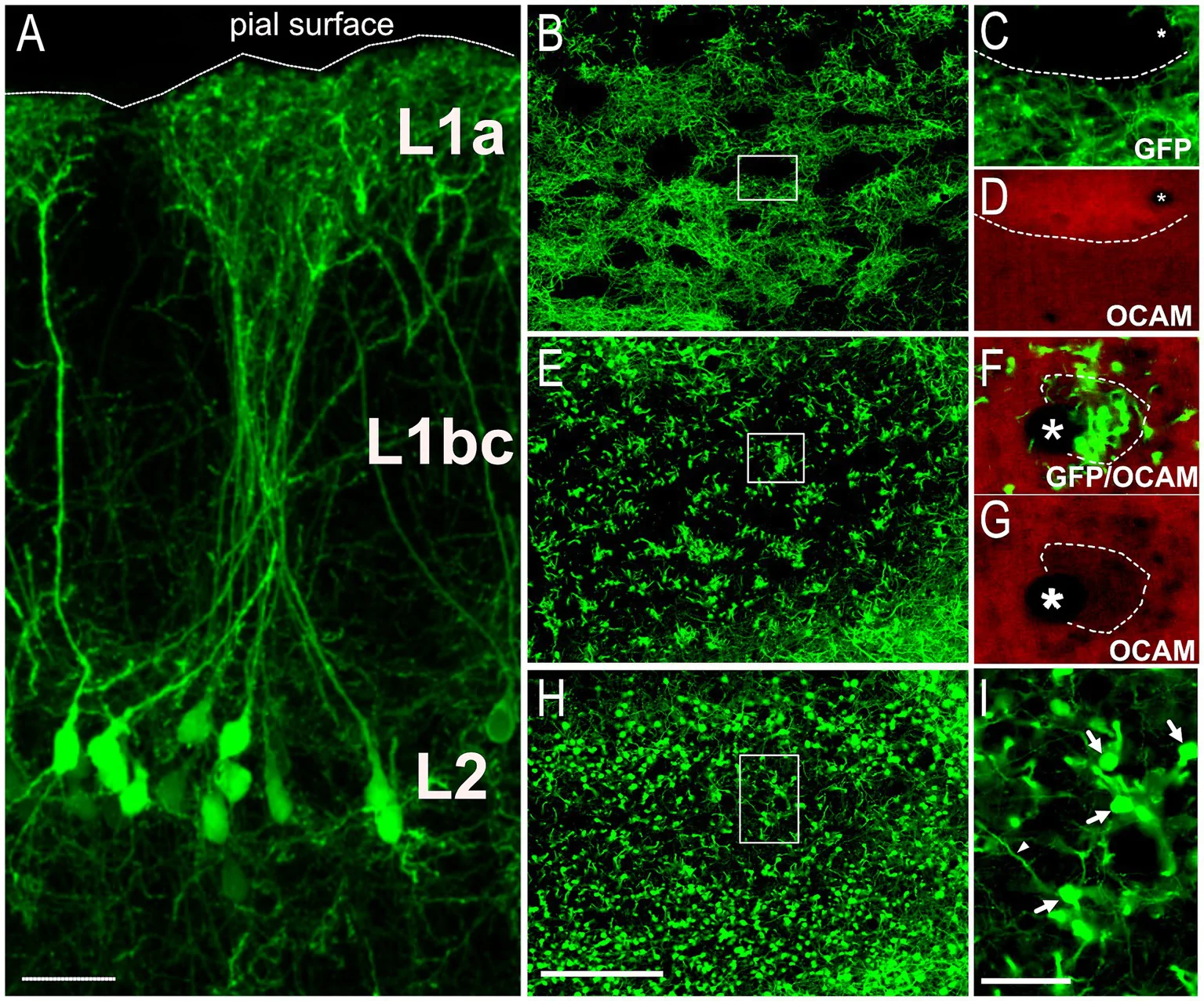
gRSC L2 neurons and dendritic bundles in L1. **(A)** A coronal section of the gRSC showed dendritic bundles visualized via *in utero* electroporation with GFP. Dendritic tufts filled sublayer L1a, whereas a prominent bundle-like organization was observed in L1b/c. Scale bar, 25 μm (**B–I**; center column). **(B,E,H)** Tangential sections (30 μm thickness) of the gRSC showing GFP-positive layer 1 dendritic bundles at the cortical surface (**B**; S7), in Layer 1b/c (**E**; S5), and near Layer 2 (**H**; S2). **(C,D)** Higher magnifications from the inset in **(B)** show complementary distribution of GFP-positive dendrites **(C)** and OCAM immunoreactivity **(D)** from S7. **(F,G)** Higher magnifications from the inset in **(E)** show complementary distribution of OCAM-negative hollow **(G)** and GFP-positive dendritic bundle in the hollow **(F)** from S5. **(I)** Higher magnification from the inset in **(H)**. Layer 2 neurons (arrows) and dendrite (arrowhead) are shown.

To further examine the tangential organization of these dendritic bundles and their relationship with OCAM-ir, I prepared serial 30-μm-thick sagittal sections from the midline of electroporated rat brains. Since the sagittal sections parallel to the midsagittal plane were tangential to the cortical surface of the gRSC, this approach allowed us to observe the tangential distribution of dendritic bundles across L1. The deepest section containing GFP-positive L2 cell bodies was defined as section 1 (S1, Supplementary Figure 2G), and serial sections toward the cortical surface were designated as S2–S7 (Supplementary Figure 2). In sections close to L2, GFP-positive cell bodies and proximal apical dendrites were observed (Figures 2H,I). In more superficial sections corresponding to L1b/c, GFP-labeled dendritic bundles were detected as discrete domains (Figures 2E,F). Additionally, sections close to the cortical surface mainly contained laterally spreading dendritic tufts in L1a (Figure 2B).

Double immunostaining for GFP and OCAM revealed a complementary distribution of GFP-labeled dendritic bundles and OCAM-ir (Figures 2F,G). OCAM-ir was weak or sparse in most superficial sections, where GFP-labeled dendritic tufts were broadly distributed (Supplementary Figure 2A). In contrast, OCAM-positive domains became prominent in sections corresponding to L1b/c, particularly from S3 to S5 (Figure 2E and Supplementary Figures 2C–E), where GFP-labeled dendritic bundles were observed within OCAM-negative domains. High-magnification images confirmed that GFP-positive dendritic bundles were surrounded by OCAM-ir, and that the two signals showed a complementary rather than overlapping distribution (Figures 2F,G and Supplementary Figure 3). This pattern was consistent with the previously reported complementary distribution of MAP2 and OCAM (Ichinohe et al., 2003). These results suggest that the MAP2-labeled dendritic bundles located within the OCAM-negative hollows in L1 of the gRSC correspond to dendritic bundles formed by the GFP-labeled L2 neurons examined in this study. This spatial relationship further suggests that OCAM-negative hollows may serve as anatomical landmarks for identifying L2 dendritic bundles.

### Ectopically expressed TrkC-dTK alters the morphology of dendritic bundles in L1 of the gRSC

To examine whether TrkC signaling is involved in the formation of dendritic bundles in L1 of the gRSC, I expressed a GFP-tagged truncated TrkC variant (TrkC-dTK-GFP) in gRSC L2 neurons via IUE. TrkC is a transmembrane receptor tyrosine kinase that activates intracellular signaling via its cytoplasmic tyrosine kinase domain upon ligand binding. A truncated form lacking this domain is expected to interfere with endogenous TrkC signaling and function as a dominant-negative receptor (Tsoulfas et al., 1993; Valenzuela et al., 1993; Joo et al., 2014). TrkC-dTK comprises amino acid residues 1–528 of TrkC. It contains neither the tyrosine kinase domain nor the TrkC. T1 isoform-specific C-terminal cytoplasmic sequence, which is known to mediate tamalin–Arf6–Rac1 signaling and induce cellular protrusions and membrane ruffling (Esteban et al., 2006). Based on my gene expression analysis and previous reports implicating TrkC signaling in synapse formation and dendritic development, I investigated the effects of TrkC-dTK overexpression. I generated a plasmid, pCAG-TrkCdTK-GFP, encoding TrkC-dTK-GFP, which is a GFP-tagged TrkC variant lacking the tyrosine kinase domain. This molecular structure closely resembles that of the TrkC. DN construct used by Joo et al. (2014), which was employed as a dominant-negative inhibitor of NT-3/TrkC signaling. I examined the subcellular localization of TrkC-dTK-GFP using confocal microscopy and confirmed that the fusion protein was distributed over most of the plasma membrane, including the membranes of the cell body and dendrites (Supplementary Figure 7).

The pCAG-TrkCdTK-GFP plasmid, or a control plasmid, pCAGGS-GFP, encoding GFP alone, was introduced into the gRSC at E18 via IUE, and the morphology of the GFP-labeled dendritic bundles was examined at P25. In both the constructs, transgene expressions were driven by the CAG promoter. In control animals expressing GFP alone, L2 dendrites formed typical dendritic bundles in L1b/c, with distinct intervening gaps and extended toward the cortical surface, where dendritic tufts spread laterally in L1a, as indicated by the arrowheads (Figures 3A,B). In contrast, the dendritic bundles remained fasciculated but were less clearly defined in animals expressing TrkC-dTK-GFP, and appeared to expand laterally at more superficial levels within L1b than in controls. The dendritic bundles displayed an inverted triangular profile in coronal sections, suggesting that TrkC-dTK expression caused dendritic bundles to adopt an expanded, inverted, cone-like morphology toward the cortical surface (Figures 3C,D).

**Figure 3:**
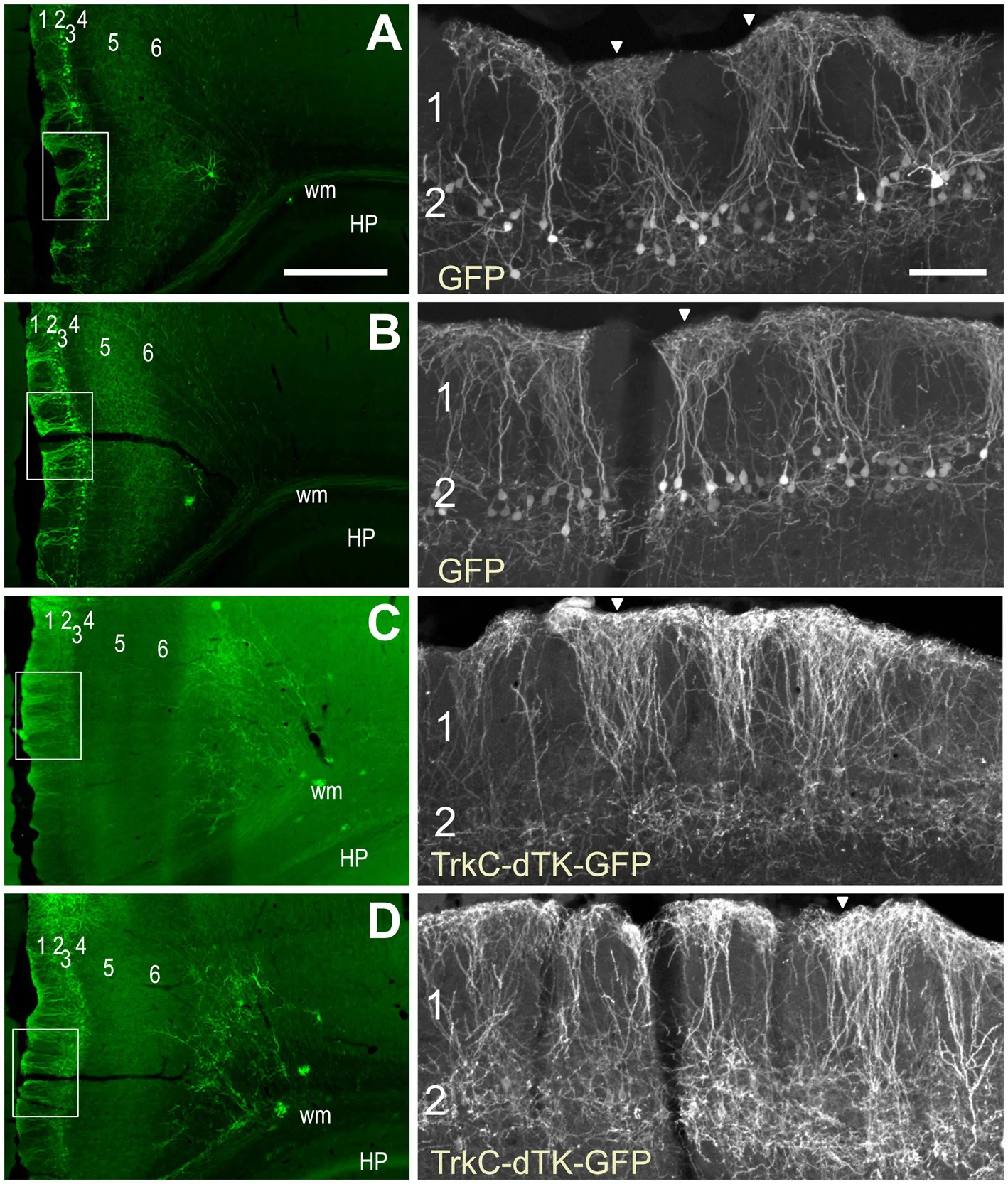
TrkC-dTK overexpression alters the morphology of dendritic bundles in L1 of the gRSC. Dendritic bundles in L1 of the gRSC were visualized via *in utero* electroporation of GFP **(A,B)** or TrkC-dTK-GFP **(C,D)**. Left column, lower-magnification images acquired with a NanoZoomer scanner (Hamamatsu). Right column, higher-magnification confocal images of the boxed regions in the left column. Expression of both transgenes was driven by the CAG promoter. Scale bars, 500 μm for right and 100 μm for left column. wm; white matter. HP; hippocampus. Numbers indicate cortical layers.

To quantify this morphological change, I analyzed the tangential cross-sectional areas of the dendritic bundles in serial sections through L1. As described above, the OCAM-negative domains surrounded by OCAM-positive regions were used as anatomical landmarks corresponding to the dendritic bundle territories (Supplementary Figure 3). Serial tangential sections corresponding to S3–S5 were examined in GFP-expressing control and TrkC-dTK-GFP-expressing animals (Figures 4A–F). These sections correspond to L1b/c, where the dendritic bundles are clearly distinguishable and are suitable for area measurements.

**Figure 4:**
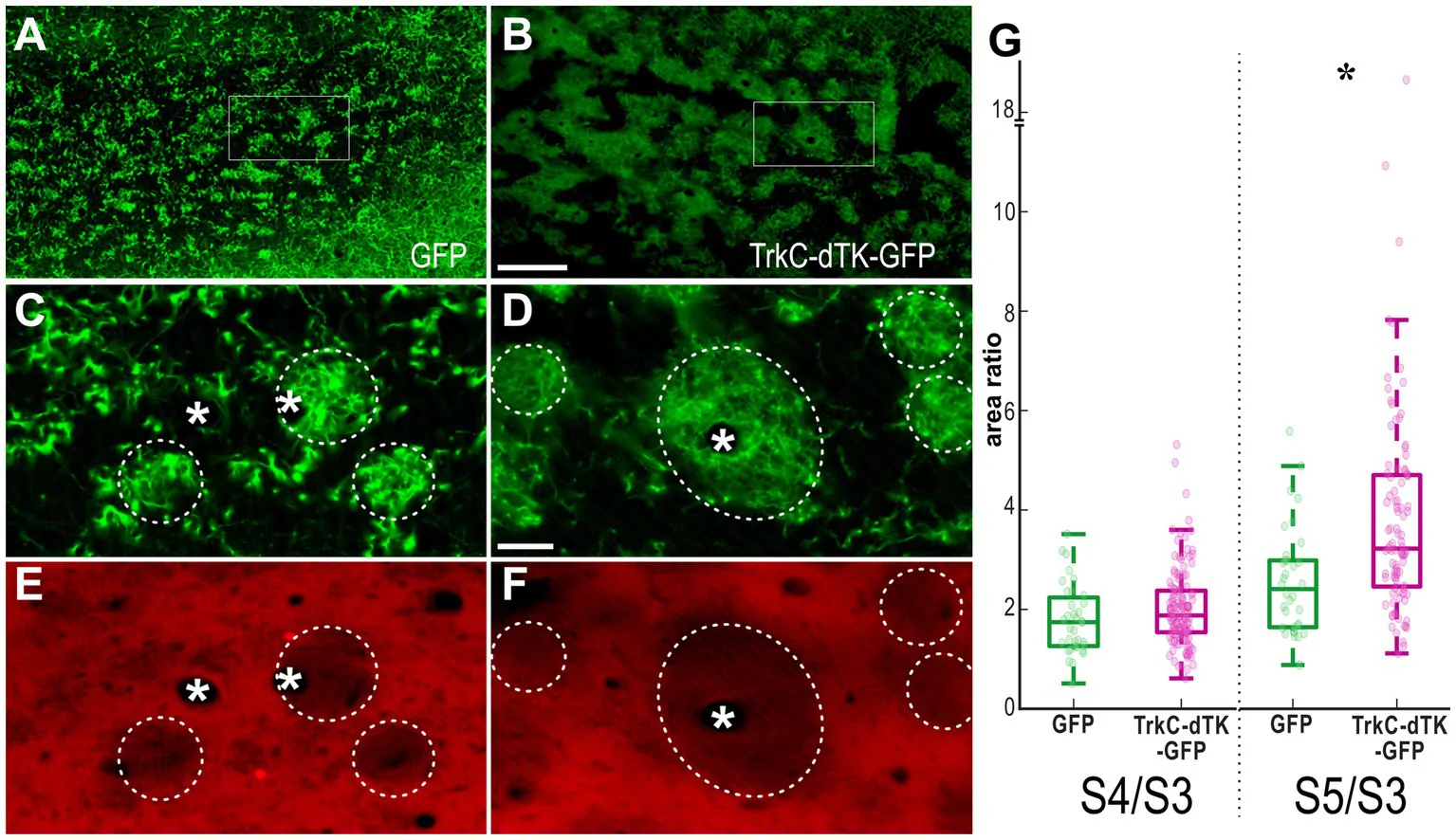
TrkC-dTK overexpression increases the area of dendritic bundles in L1 of the gRSC. Tangential sections (30 μm thick) of the gRSC show dendritic bundles and OCAM immunoreactivity at S5. **(A,C,E)** Control. **(B,D,F)** TrkC-dTK overexpression. **(A,B)** Lower-magnification images showing GFP-positive dendritic bundles. **(C,D)** Higher-magnification images of the boxed regions in **(A,B)**. **(E,F)** OCAM immunoreactivity in the same specimens shown in **(C,D)**. Scale bars, 250 μm in **(B)** for **(A,B)**, and 50 μm in **(D)** for **(C–F)**. **(G)** Distributions of dendritic bundle area ratios in tangential sections are shown as box plots with individual data points. For each bundle, the area measured at S4 or S5 was normalized to that at S3, and the resulting S4/S3 and S5/S3 ratios were compared between the control and TrkC-dTK groups. Dendritic bundle areas were analyzed at P25. Sample sizes: S4/S3, control = 35 bundles from 4 rats, TrkC-dTK = 135 bundles from 13 rats; S5/S3, control = 34 bundles from 4 rats, TrkC-dTK = 97 bundles from 10 rats. Asterisks indicate significant differences determined by two-sided Welch’s t-tests on log-transformed data with Holm correction (S4/S3, *p* = 0.0938; S5/S3, *p < 0.001).

I analyzed 35 dendritic bundles from four GFP-expressing control gRSC hemispheres and 135 dendritic bundles from 13 TrkC-dTK-GFP-expressing gRSC hemispheres. The mean cross-sectional areas of the dendritic bundle territories in S3 and S4 did not differ significantly between the control and TrkC-dTK-GFP-expressing samples. In contrast, the area in S5 showed an approximate 1.3-fold increase in TrkC-dTK-GFP-expressing samples compared with the controls (Supplementary Figure 4).

Next, I examined how the bundle area changed along the depth axis of L1 by calculating the ratios of the areas at S4 and S5 relative to S3. In both the control and TrkC-dTK-GFP-expressing samples, the bundle area increased from S3 to S4, with the S4/S3 ratio being approximately two-fold in both groups. However, the expansion from S3 to S5 was greater in TrkC-dTK-GFP-expressing dendritic bundles than in the controls. The mean S5/S3 area ratio was significantly greater in the TrkC-dTK-GFP-expressing samples (3.84) than in the controls (2.56) (Figure 4G).

These results indicate that the TrkC-dTK alters the three-dimensional morphology of dendritic bundles in gRSC L1. In particular, TrkC-dTK-GFP expression preferentially expanded the dendritic bundle territories in the more superficial part of L1b/c, causing the bundles to expand toward the cortical surface and adopt an inverted cone-like configuration. Thus, TrkC signaling appears to contribute to the proper structure of L2 dendritic bundles within the modular architecture of gRSC L1.

### TrkC-dTK expression increases dendritic branching of gRSC L2 neurons

The expansion of the dendritic bundle regions caused by TrkC-dTK-GFP expression suggests that the morphology of individual L2 dendrites may be altered by the inhibition of TrkC signaling. The gRSC L2 pyramidal neurons have a characteristic dendritic morphology that is distinct from that of pyramidal neurons in other neocortical regions. In particular, their apical dendrites extend through L1 with relatively few branches or oblique dendrites, and extensive branching is mainly observed near the cortical surface at L1a (Wyss et al., 1990; Ichinohe et al., 2008; Kurotani et al., 2013). To examine whether TrkC-dTK-GFP expression affects the morphology of individual L2 neurons, I performed sparse single-cell labeling using the Cre-loxP system delivered by IUE.

A plasmid driving Cre-dependent mCherry expression was introduced along with a diluted Cre-expressing plasmid, allowing a small subset of L2 neurons to be labeled with mCherry. Simultaneously, GFP or TrkC-dTK-GFP was expressed in gRSC L2 neurons via IUE as described above. This approach enabled us to visualize the overall dendritic bundle structure using GFP fluorescence, and simultaneously trace the dendritic morphology of individual mCherry-labeled neurons (Figure 5).

**Figure 5:**
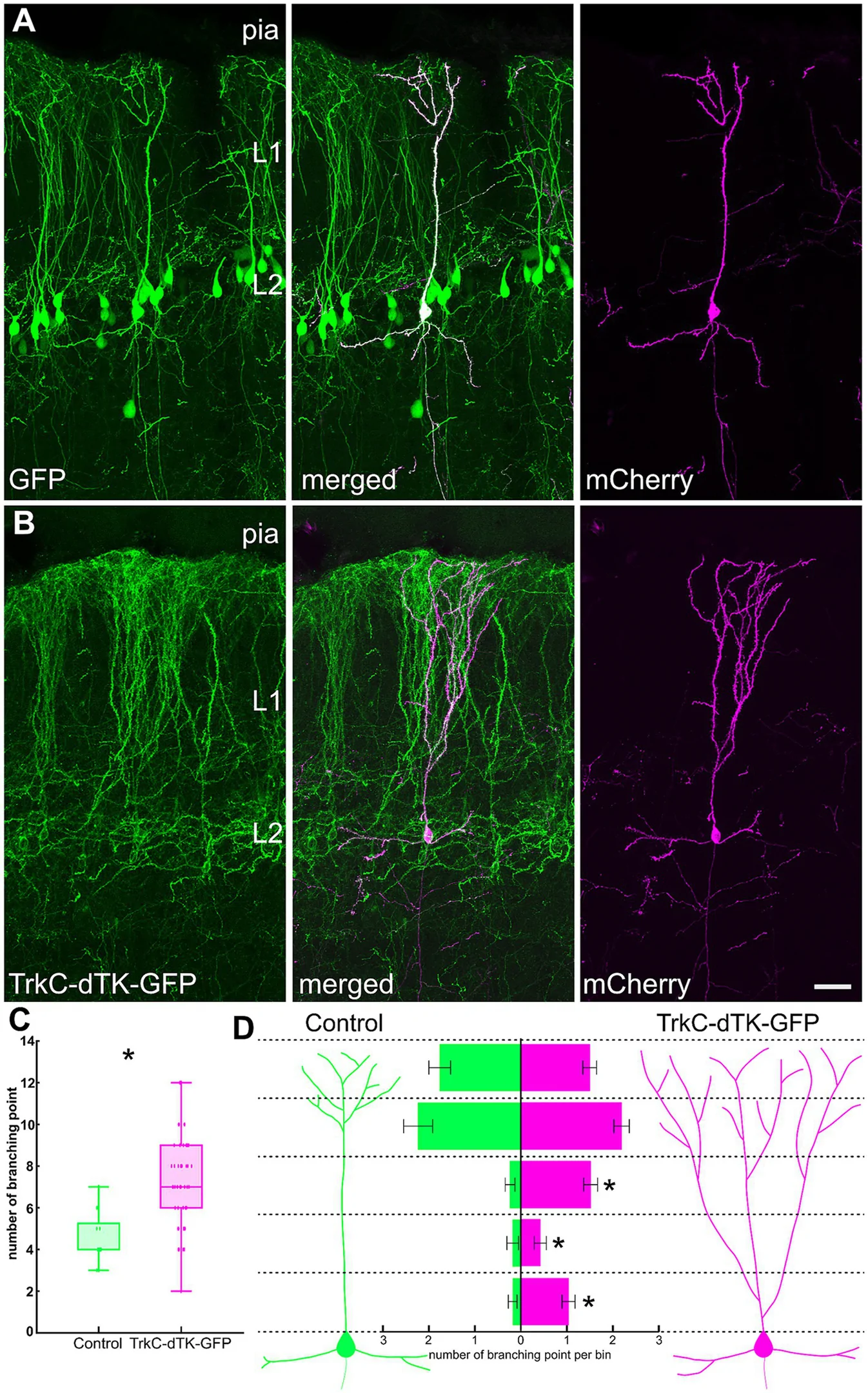
Single-cell labeling and quantification of dendritic morphology in L1 of the gRSC. Dendritic bundles in L1 of the gRSC were visualized in GFP-expressing control samples **(A)** and TrkC-dTK-GFP-expressing samples **(B)**. Sparse labeling with mCherry was used to visualize the dendritic morphology of individual neurons. Left column: GFP-labeled dendritic bundles; middle column: merged images; right column: mCherry-labeled single neurons. Scale bar, 20 μm. **(C)** Quantification of the total number of dendritic branch points in control and TrkC-dTK-GFP-expressing L2 neurons of the gRSC based on mCherry single-cell labeling. **(D)** Schematic representation and quantitative analysis of dendritic branch points across L1. L1 was divided into five depth bins parallel to the cortical surface, with bin 1 corresponding to the deepest region and bin 5 to the most superficial region. The number of branch points in each bin was compared between control and TrkC-dTK-GFP-expressing neurons. Neurons were analyzed at P25. Sample sizes: control = 17 neurons from 4 rats; TrkC-dTK = 52 neurons from 4 rats. Statistical significance was determined by two-sided Mann–Whitney U tests. Bonferroni correction was applied to the five comparisons in the bin-wise analysis. *p < 0.001.

In the GFP-expressing control samples, sparsely labeled mCherry-positive L2 neurons showed the characteristic dendritic morphology of gRSC L2 pyramidal cells. Their apical dendrites extended toward the cortical surface, with relatively few branches within L1, consistent with previous reports (Figure 5A and Supplementary Figure 5). In contrast, mCherry-labeled L2 neurons in TrkC-dTK-GFP-expressing samples exhibited more frequent branching along their apical dendrites (Figure 5B and Supplementary Figure 6).

For quantitative analysis, I selected sparsely labeled mCherry-positive neurons whose dendrites could be traced without overlapping with dendrites from neighboring labeled cells. Confocal z-stack images were used to count the dendritic branch points in individual neurons. I analyzed 17 neurons from four GFP-expressing control animals and 52 neurons from four TrkC-dTK-GFP-expressing animals. The dendritic morphology was generally similar within each group, although some variability was observed. Overall, control neurons showed apical dendrites with few branches in L1b/c and dendritic tufts in L1a. In contrast, mCherry-labeled TrkC-dTK-expressing neurons appeared to show more dendritic branches, some of which extended laterally (e.g., Supplementary Figure 7A). Because the sections used in this study were only 40 μm thick, it was difficult to analyze the entire dendritic arbor, including the basal dendrites. Therefore, the present analysis focused specifically on the apical dendrites. The number of dendritic branch points was significantly higher in TrkC-dTK-GFP-expressing neurons than in the control. Mean number of branch points per neuron was 4.59 in control neurons and 7.33 in TrkC-dTK-GFP-expressing neurons (Figure 5C).

To determine where the increase in branching occurred along the apical dendrite, I divided the L1 portion of each traced dendrite into five depth bins as described in the methods section. I then counted the number of branch points within each bin and compared the distribution between control and TrkC-dTK-GFP-expressing neurons.

In control neurons, only a small number of branch points were observed in the deeper regions of L1. In contrast, TrkC-dTK-GFP-expressing neurons showed an increased number of branch points in the proximal-to-middle portions of L1, particularly in bins 1–3. In contrast, the number of branch points in the more superficial bins, corresponding to the distal dendritic region near L1a, did not show a clear increase in TrkC-dTK-GFP-expressing neurons compared to controls (Figure 5D).

These results indicate that the inhibition of TrkC signaling increases dendritic branching in gRSC L2 neurons, particularly in the deeper regions of L1 near the cell body. This increase in proximal dendritic branching may contribute to the expansion of dendritic bundle territories observed in the TrkC-dTK-GFP-expressing samples. Because this analysis was performed using 40-μm-thick sections and was restricted to dendrites that could be continuously traced from the cell body, distal dendritic branches near the cortical surface may have been underestimated.

## Discussion

In this study, I examined the role of NT-3-related signaling in the formation of dendritic bundles in L2 neurons of the gRSC. We have previously reported that NT-3 is strongly and transiently expressed in the gRSC L2 before dendritic bundle formation (Miyashita et al., 2010). Here, I focused on TrkC, a receptor for NT-3, and used TrkC-dTK-GFP, a TrkC variant lacking the intracellular tyrosine kinase domain, as a putative dominant-negative form to perturb TrkC-dependent signaling in the developing gRSC L2 neurons.

### TrkC-dependent regulation of dendritic branching and bundle morphology in gRSC L2 neurons

Overexpression of TrkC-dTK-GFP altered the morphology of dendritic bundles in L1. In particular, the dendritic bundle territories expanded toward the superficial part of L1, resulting in an inverted cone-like configuration. Sparse single-cell labeling further showed that individual TrkC-dTK-GFP-expressing L2 neurons exhibited increased dendritic branching, especially in the proximal-to-middle portions of L1. As shown in the control experiments, the apical dendrites of gRSC L2 neurons normally extend relatively straight toward the pia with few branches, and several neighboring dendrites fasciculate to form a dendritic bundle. TrkC-dTK expression increases the number of obliquely oriented dendritic branches in the proximal portion of the apical dendrite. As these laterally extending branches overlap with neighboring dendrites, they may broaden the territory occupied by the dendritic bundle toward the pial surface. These findings suggest that TrkC-dependent signaling contributes to the organization of gRSC L2 dendritic bundles by regulating the branching patterns of individual apical dendrites.

Our expression analysis provided an important context for the interpretation of this phenotype. NT-3 expression was the highest in gRSC L2 at P3, before dendritic bundle formation (Miyashita et al., 2010), whereas TrkC expression was maintained at above-average levels and was relatively elevated in gRSC L2 at P12, after dendritic bundle formation. Thus, within the examined developmental stages, NT-3 and TrkC exhibited partially complementary temporal expression patterns. This observation suggests that NT-3 and TrkC may not simply act as a ligand-receptor pair at a single developmental time point. Instead, they may contribute to gRSC L2 specialization through temporally distinct but functionally related mechanisms.

Neurotrophins and their receptors regulate multiple aspects of neuronal differentiation, including dendritic growth, branching, spine formation, synaptic development, and cell–cell competition (McAllister et al., 1995, 1997, 1999; Schinder and Poo, 2000; Takahashi et al., 2011; Joo et al., 2014; Ammendrup-Johnsen et al., 2015; Naito et al., 2017; Viale et al., 2019). Their effects on dendritic morphology are highly context-dependent and can vary according to the neuronal subtype, cortical layer, developmental stage, and brain region. For example, in the ferret visual cortex, the basal dendrites of layer IV pyramidal neurons respond strongly to BDNF, whereas neurons in deeper layers respond more prominently to NT-4 (McAllister et al., 1995). In the retrosplenial cortex, NT-3 regulates the length and branching of apical dendrites without markedly affecting basal dendrites (Viale et al., 2019). Neurotrophins can also exert opposing effects on the same cortical area. In the visual cortex, BDNF promoted dendritic growth in layer IV, but inhibited it in layer VI, whereas NT-3 showed the opposite pattern. Such push-pull mechanisms may allow neurotrophin signaling to shape layer-specific dendritic architectures (McAllister et al., 1997).

Different receptor isoforms may further diversify the effects of neurotrophin signaling. In the case of TrkB, full-length TrkB and truncated TrkB. T1 regulate distinct dendritic compartments, with full-length TrkB preferentially promoting proximal branching and TrkB. T1 contributing to distal dendritic extension (Yacoubian and Lo, 2000). In the cerebellum, TrkC signaling has been implicated in the competitive regulation of Purkinje cell dendritic development. The deletion of TrkC from all Purkinje cells does not necessarily cause a marked morphological defect, whereas the deletion of TrkC in only a subset of cells leads to shortened dendrites in knockout cells (Joo et al., 2014). This suggests that dendritic development depends on the relative rather than absolute levels of neurotrophin signaling among neighboring neurons. In contrast, the inhibition of TrkC signaling in the opossum cerebellum increases dendritic branching, possibly through the altered availability of NT-3 for other receptors, such as p75NTR (Tepper et al., 2020). These findings indicate that the effect of TrkC signaling on dendritic morphology is not uniform but depends strongly on the cellular and developmental context.

Our results are consistent with this context-dependent view of TrkC function. In gRSC L2 neurons, TrkC-dTK-GFP overexpression increased the number of apical dendritic branch points and altered the shape of dendritic bundles. This phenotype differed from the reduction in dendritic length and branching reported in previous NT-3 knockdown experiments (Viale et al., 2019). This suggests that TrkC-dependent signaling normally helps to restrict excessive branching in specific regions of the apical dendrite. gRSC L2 neurons typically extend relatively unbranched apical dendrites through L1 before forming distal tufts near the cortical surface, L1a. Therefore, premature or ectopic perturbation of TrkC-dependent signaling may disrupt this compartmentalized dendritic pattern and induce excessive branching in regions where branching is normally limited.

### Potential roles of TrkC isoforms and calcium signaling

One possible mechanism underlying this effect is the involvement of different TrkC isoforms during the distinct phases of gRSC L2 dendritic development. In our GeneChip analysis, the *trkC* probe set corresponding to the 5′ region showed a modest increase in gRSC L2 at P12 compared with the probe set corresponding to the 3′ region. Since the 3′ region includes sequences encoding the intracellular tyrosine kinase domain, this pattern raises the possibility that truncated TrkC isoforms lacking the tyrosine kinase domain may be relatively increased during later stages of dendritic bundle maturation. However, this interpretation remains speculative because differences between the probe sets do not directly demonstrate the expression of specific TrkC isoforms. Isoform-specific RT-PCR, RNA sequencing, and quantitative *in situ* hybridization are required to determine whether truncated TrkC isoforms are expressed in gRSC L2 at P12. Nevertheless, the phenotype induced by TrkC-dTK-GFP overexpression suggests that the balance between full-length and kinase-deficient TrkC signaling influences the branching pattern of gRSC L2 apical dendrites.

Our pathway-level analysis further suggests that calcium signaling may be associated with the molecular state of gRSC L2 at P12. Among the genes that contributed to the separation of gRSC L2 at P12 in the neurotrophin signaling pathway dataset (rno04722), several were associated with the calcium signaling pathway. Calcium signaling is broadly used in many cellular processes and is therefore not specific to gRSC L2. However, the enrichment of calcium signaling-related genes among neurotrophin pathway components suggests that the intracellular response to neurotrophins may differ according to the cortical area, cortical layer, and developmental stage. Such differences in downstream signaling could help explain why neurotrophins exert distinct and sometimes opposing effects on dendritic morphology in different neuronal populations.

### Local actions of NT-3/TrkC signaling during dendritic bundle formation

An important question is how NT-3/TrkC signaling acts in gRSC L2 cells during dendritic bundle formation. Neurotrophins can act as retrograde signals from target cells to afferent neurons in several neural circuits. For example, NT-3, expressed in layer 4 of the visual cortex, has been implicated in the development of thalamocortical circuits (Ma et al., 2002). Since NT-3 expression in the gRSC occurs during the establishment of afferent circuits, NT-3 in gRSC L2 could potentially contribute to circuit formation through a retrograde signaling mechanism. However, neurotrophin signaling can also operate through local or anterograde mechanisms, depending on the cellular context. For example, in the cerebellum, NT-3, derived from granule cells influences Purkinje cell dendritic development through TrkC-dependent signaling (Joo et al., 2014). Thus, the function of NT-3/TrkC signaling in gRSC should be considered in terms of local cellular and circuit contexts.

A previous study showed that the knockdown of NT-3 by shRNA reduced apical dendrite length and the number of branch points in P7 rat gRSC neurons (Viale et al., 2019). Since NT-3 was reduced in NT-3-expressing cells themselves, these findings suggest that NT-3 in gRSC L2 may not be explained solely by a classical retrograde or anterograde neurotrophin signaling model. Instead, NT-3 may regulate dendritic development through autocrine-like or local paracrine mechanisms, acting within the same L2 neuronal population. Consistent with this idea, our previous study showed that ectopic NT-3 expression in the S1 barrel field induces dendritic bundle formation in a largely cell-autonomous manner, while also partially affecting the neighboring cells (Miyashita et al., 2010). These observations suggest that NT-3 acts locally to regulate dendritic development in gRSC L2 neurons.

### Distinct effects of NT-3 knockdown and TrkC perturbation

The apparent difference between the previous NT-3 knockdown experiments and the present TrkC-dTK-GFP overexpression experiment may reflect several factors. In the NT-3 shRNA experiment, apical dendrite length and branching were reduced (Viale et al., 2019), whereas in the present study, perturbation of TrkC-dependent signaling increased dendritic branching. First, the developmental timing of the analysis differed between the studies. Viale et al. analyzed neurons at P7, when dendritic bundles are still forming, whereas I analyzed neurons at P25, when dendritic bundle morphology is more clearly established. Secondly, ligand knockdown and receptor perturbation may not have similar effects. Reducing NT-3 expression may impair early dendritic growth, whereas disrupting TrkC-dependent signaling at later stages may alter the refinement or restriction of dendritic branching. Third, our analysis focused not only on individual dendrites, but also on the organization of dendritic bundles within L1, which may reveal later changes in bundle architecture that are not apparent at earlier stages. Future analyses of sparsely labeled neurons at earlier developmental stages, such as P7, using the same approach as used in the present study, would help clarify how NT-3 and TrkC signaling contribute to the transition from initial dendritic growth to mature dendritic bundle formation.

### NT-3/TrkC signaling and the modular organization of gRSC L1

The present findings also raise the possibility that NT-3- and TrkC-related signaling contribute to the modular organization of gRSC L1. Dendritic bundles in the gRSC are formed by the apical dendrites of L2 neurons. gRSC L1 contains these L2 dendritic bundles as well as OCAM-positive dendritic tufts of L5 neurons, which are distributed in complementary domains (Ichinohe et al., 2003). These modular postsynaptic structures are associated with distinct presynaptic inputs. Previous studies have shown that dendritic bundle domains are associated with vGluT2-positive thalamocortical terminals in the anterodorsal and anteroventral thalamic nuclei, whereas OCAM-positive domains are associated with vGluT1-positive corticocortical terminals (Ichinohe, 2012). Thus, the gRSC L1 is not simply a layer containing bundled dendrites; rather, it contains a higher-order modular organization in which the postsynaptic dendritic compartments and presynaptic input domains are aligned.

In this context, NT-3 expressed by gRSC L2 neurons may contribute not only to dendritic development but also to the establishment of local circuit architecture. One possibility is that NT-3 secreted from postsynaptic gRSC L2 neurons acts locally to influence the organization of afferent terminals, including the thalamocortical and corticocortical inputs. For example, NT-3 may promote the localization or stabilization of thalamocortical inputs within dendritic bundle domains, while indirectly contributing to the segregation of corticocortical inputs into OCAM-positive domains. If NT-3 is involved not only in the formation of dendritic bundles but also in the formation of local functional microcircuits in gRSC L1, elucidating the mechanisms underlying the formation of these local microcircuits would be of considerable significance. However, as the present study did not examine presynaptic markers after TrkC-dTK-GFP expression, this possibility remains speculative.

TrkC-dTK, a putative TrkC signal inhibitor, expression altered the morphology of dendritic bundles; however, the phenotype was not uniform along the depth axis of L1. In TrkC-dTK-GFP-expressing gRSC L1, dendritic bundles expanded preferentially toward the superficial part, resulting in an inverted cone-like configuration. Importantly, the proximal portions of the dendrites remained bundled, whereas the distal portions showed abnormal expansion. This phenotype suggests that TrkC-dependent signaling may not be required for the initial fasciculation of apical dendrites near L2, but may instead contribute to the refinement of dendritic morphology and the maintenance of bundle architecture in more superficial regions of L1. It has been suggested that the impaired dendritic architecture of a subset of neurons in gRSC L1 is sufficient to produce abnormal behaviors in spatial exploration and/or navigation and spatial memory (Viale et al., 2019). These results suggest that the dendritic bundle is a possible functional unit of gRSC. It is important to determine whether such changes in dendritic bundle morphology caused by the inhibition of TrkC signaling are accompanied by corresponding changes in the distribution of vGluT2-positive thalamocortical terminals or vGluT1-positive corticocortical terminals. Characterizing this microcircuit architecture is crucial for determining if dendritic bundles represent the basic functional cortical unit.

### A developmental model for NT-3/TrkC-dependent organization of gRSC L2

The organization of the gRSC L2 may also be related to the dense packing of neurons in this layer. gRS L2, also known as the outer granular layer, is characterized by a dense packing of small neurons, distinct from neocortical L2. Interestingly, several cortical regions or layers with relatively dense granular organization, such as L4 of the primary visual cortices and L2/3 of the entorhinal cortex, also show strong NT-3 expression during development (Vigers et al., 2000). This suggests that NT-3 expression is associated with the formation of densely packed granular or module-like cortical structures. In our previous study, ectopic NT-3 expression in the S1 barrel field induced dendritic bundle formation and affected the local arrangement of labeled cells. Specifically, the cells seemed to cluster closely together and appeared more densely packed than the control cells expressing GFP (Figures 4A,B from Miyashita et al., 2010). Although this observation was not quantitatively analyzed, it suggests that NT-3 may influence not only dendritic morphology, but also the local organization of neurons.

Taken together, our findings suggest a model in which NT-3 and TrkC contribute to different but related aspects of gRSC L2 specialization, such as dendritic bundle formation. Early postnatal NT-3 expression may promote the positioning or local organization of gRSC L2 neurons, and support the initial growth and alignment of their apical dendrites. Subsequently, TrkC-dependent signaling may help refine the dendritic morphology by restricting excessive branching and maintaining the modular organization of dendritic bundles in L1. Rather than acting as a simple ligand-receptor pair at a single developmental stage, NT-3 and TrkC may participate in a temporally coordinated signaling system that shapes the distinctive dendritic and afferent architecture of the gRSC. Further studies are required to determine how full-length and truncated TrkC isoforms, calcium-dependent downstream pathways, and afferent inputs interact to establish the modular organization of gRSC L1.

## Data Availability

The GeneChip data analyzed in this study are publicly available in the NCBI Gene Expression Omnibus (GEO) under accession number GSE346953: https://www.ncbi.nlm.nih.gov/geo/query/acc.cgi?acc=GSE346953.

## Supplementary material

SUPPLEMENTARY FIGURE 1 Developmental dynamics of TrkC expression in the gRSC. **(A)** The same expression data as in Figure 1A are shown as z-score heat maps calculated across the eight 694 GeneChip arrays. Mean expression values from two biological replicates are shown. Mean values of the two 695 probe sets are presented. **(B)** Normalized RMA expression values for two TrkC probe sets under 12 696 experimental conditions.

SUPPLEMENTARY FIGURE 2 Complementary distribution of GFP-labeled dendritic bundles and OCAM immunoreactivity in serial tangential sections of the gRSC. Serial tangential sections (30 μm thick) of the gRSC were arranged from the cortical surface **(A)** to layer 2 **(G)**. Left column: GFP-labeled dendritic bundles; middle column: merged images; right column: OCAM immunoreactivity. Plus signs in **(A)** indicate OCAM-positive domains in which GFP-labeled dendritic bundles are absent. Asterisks in **(B)** indicate GFP-labeled dendritic bundles located within OCAM-negative domains. The X in the middle image of **(G)** indicates the blood vessel shown at higher magnification in the inset. Each section was 30 μm thick; the seven serial sections span 210 μm, covering layer 1 and extending into layer 2. The deepest section containing L2 cells (inset in **G**) was defined as section 1 (S1), followed by S2–S7 toward the cortical surface. Scale bars, 250 μm for the low-magnification images and 50 μm for the inset in **(G)**.

SUPPLEMENTARY FIGURE 3 Dendritic bundles and OCAM immunoreactivity. **(A)** Schematic illustration of tangential planes through gRSC L1. L2 neurons and their apical dendrites, which form bundles extending through OCAM-negative domains, are shown. **(B)** Tangential section of gRSC L1 after in utero electroporation of GFP. GFP-labeled dendritic bundles are shown in the top panel, OCAM immunoreactivity in the bottom panel, and the merged image in the middle panel. The area enclosed by the dotted line indicates an OCAM-negative region that overlaps with the dendritic bundle domain.

SUPPLEMENTARY FIGURE 4 Comparison of dendritic bundle area between control and TrkC-dTK-expressing animals. Bundle areas at S3 (left), S4 (center), and S5 (right) were compared between the control and TrkC-dTK groups. Values were normalized to the mean of the corresponding control group. Error bars indicate SD, and circles represent individual data points. Sample sizes: S3 and S4, control = 35 bundles from 4 rats, TrkC-dTK = 135 bundles from 13 rats; S5, control = 34 bundles from 4 rats, TrkC-dTK = 97 bundles from 10 rats. Statistical comparisons were performed using two-sided Welch’s t-tests on log-transformed data with Holm correction for the three comparisons. Adjusted *p* values: S3, *p* = 0.369; S4, *p* = 0.147; S5, *p* = 0.0253.

SUPPLEMENTARY FIGURE 5 Six examples of mCherry-labeled control gRSC L2 neurons.

SUPPLEMENTARY FIGURE 6 Six examples of mCherry-labeled TrkC-dTK-expressing gRSC L2 neurons.

SUPPLEMENTARY FIGURE 7 Subcellular localization of TrkC-dTK-GFP protein. **(A,D,G)** Sparse labeling with mCherry of TrkC-dTK-expressing gRSC L2 neurons following in utero electroporation. Note that some dendritic branches extend laterally in **(A)**. **(B,E,H)** Merged images. **(C,F,I)** TrkC-dTK-GFP signals. **(A–C)** Low-magnification images. **(D–F)** Higher-magnification images of 733 the region indicated by the arrowhead. **(G–I)** Higher-magnification images of the region indicated by the arrow.
